# Effect of the underlayer on the elastic parameters of the CoFeB/MgO heterostructures

**DOI:** 10.1038/s41598-024-71110-1

**Published:** 2024-08-31

**Authors:** S. Shekhar, S. Mielcarek, Y. Otani, B. Rana, A. Trzaskowska

**Affiliations:** 1 Faculty of Physics, Institute of Spintronics and Quantum Information, Adam Mickiewicz University, Uniwersytetu Poznańskiego 2, 61-614 Poznan, Poland; 2 Center for Emergent Matter Science, RIKEN, 2-1 Hirosawa, Wako, 351-0198 Japan; 3 Institute for Solid State Physics, University of Tokyo, Kashiwa, Chiba 277-8581 Japan

**Keywords:** Brillouin light scattering, CoFeB thin film, Surface acoustic waves, Elastic parameters, Underlayer material, Surfaces, interfaces and thin films, Mechanical properties, Spintronics

## Abstract

We investigated the thermally induced surface acoustic waves in CoFeB/MgO heterostructures with different underlayer materials. Our results show a direct correlation between the density and elastic parameters of the underlayer materials and the surface phonon dispersion. Using finite element method-based simulations, we calculate the effective elastic parameters (such as elastic tensor, Young’s modulus, and Poisson’s ratio) for multilayers with different underlayer materials. The simulation results, either considering the elastic parameters of individual layers or considering the effective elastic parameters of whole stacks, exhibit good agreement with the experimental data. This study will help us deepen our understanding of phonon properties and their interactions with other quasiparticles or magnetic textures with the help of these estimated elastic properties.

## Introduction

Rayleigh surface acoustic waves (SAWs) are characterized by the collective oscillatory motion of lattice structures around their equilibrium positions, propagating along the surface of an elastic medium^1^. These SAWs consist of both longitudinal and transverse waves, exhibiting elliptical polarization in the sagittal plane. In anisotropic media, the phase and group velocities of SAWs are influenced by the relative orientation of the propagation direction to the crystallographic axes, increasing the complexity of their analytical description^2^.

SAWs have diverse applications across various fields due to their high sensitivity, low power consumption, and seamless integration with integrated circuit technology. They have been utilized for biosensor application^3^, material characterization for nondestructive testing^2^, structural health monitoring^4^, lab-on-chip application^5^, fluid manipulation purposes^6^ and in telecommunications^7,8^.

Numerous experimental investigations have explored various aspects, including determining nonlinear elastic properties at the interface between rough surfaces of solids^9^ and determining the elastic properties of thin films^10–12^, demonstrating the wide applicability of these waves in different material systems. SAWs offer a non-invasive and highly sensitive method for the precise measurement of elastic parameters. Particularly in the realm of magnetic thin films, the study of SAWs provides insights into interactions such as magnon–phonon interactions^13,14^, phonon–skyrmion interactions^15^, and phonon–spin current interactions^16,17^, which are pivotal for the advancement of multifunctional spintronics devices.

CoFeB/MgO heterostructures stand out as promising candidates for future spintronics devices^18–20^. In these structures, the underlayer, acting as a buffer between the substrate and the CoFeB layer, plays a crucial role in determining various interfacial properties, such as the Dzyaloshinskii–Moriya interaction (DMI)^21^, perpendicular magnetic anisotropy (PMA)^22^, voltage-controlled magnetic anisotropy^23,24^, Gilbert damping^25^ and ultrafast spin dynamics^26^. Careful selection of underlayer materials enables tailored properties suitable for specific spintronics applications. Moreover, the underlayer material significantly impacts the elastic properties of the heterostructures, including Young’s modulus and Poisson’s ratio, thereby influencing phonon dispersion and interactions with other quasiparticles, such as magnons and skyrmions.

In this study, we investigated thermally excited SAWs in Si/SiO_2_/X/CoFeB/MgO/Al_2_O_3_ heterostructures with various underlayer materials (X) using Brillouin light spectroscopy (BLS). Additionally, numerical simulations utilizing finite element method-based COMSOL Multiphysics software were conducted to validate our experimental results and estimate elastic parameters such as Young’s modulus and Poisson’s ratio for the CoFeB/MgO heterostructures.

## Materials and methods

### Sample details

**Fig. 1:**
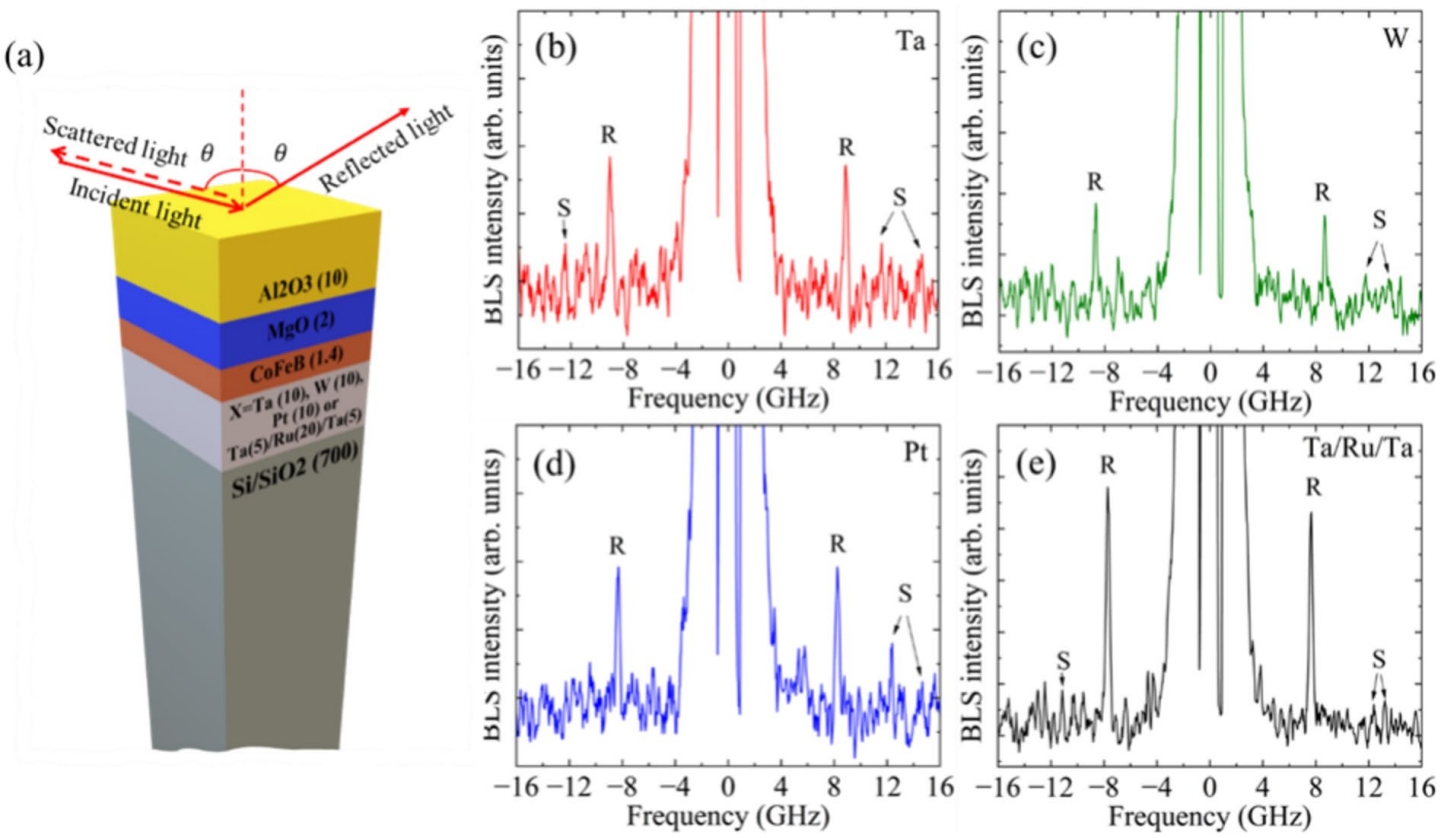
(**a**) Schematic of the studied samples and geometry of the incident reflected and scattered laser beams used for the measurements. (**b**–**e**) Observed BLS spectra for the Si/SiO_2_/X/CoFeB/MgO/Al_2_O_3_ samples corresponding to the wavevector *q* = 16.5 µm^−1^ collected for 12 h, where X represents the different underlayer materials indicated in the top right-hand corner.

We investigated multilayer thin films of X/Co_20_Fe_60_B_20_(1.4)/MgO(2)/Al_2_O_3_(10) grown on a Si/SiO_2_(700) substrate with various underlayer materials (X) (see Fig. 1a). Here, X represents Ta (10), Pt (10), W (10) or Ta (5)/Ru (20)/Ta (5), with the numbers in parentheses indicating the thickness of the layers in nanometers. The Co_20_Fe_60_B_20_ layer is referred to as CoFeB throughout the manuscript. The CoFeB thickness is intentionally maintained at 1.4 nm to observe significant variations in the interfacial properties with different underlayer materials, which will be beneficial for our future studies of magnons and their interactions with phonons. These multilayer structures are deposited on a thermally oxidized Si (001) substrate by radio-frequency (rf) sputtering at a base pressure of 10^–8^ Torr at room temperature. The top Al_2_O_3_ layer acts as a protective layer for MgO and thus the CoFeB/MgO interface, safeguarding the multilayers from degradation caused by moisture. Subsequently, the deposited films undergo annealing at 280 °C under vacuum for 1 h under a perpendicular magnetic field of 600 mT. Additional information regarding the sample fabrication process can be found in Refs.^27,28^.

### Experimental setup

The thermally excited SAWs in CoFeB/MgO heterostructures were studied using a six-pass tandem BLS (JRS Scientific Instruments), providing a contrast of 10^15,29,30^. A single-mode Nd:YAG diode-pumped laser emitting a second harmonic at a wavelength of ${\lambda }_{0}=532$ nm with an output power of 200 mW (Excelsior, Spectra Physics) served as the light source. All measurements were conducted at room temperature in the backscattering geometry with *pp* polarization of light. Detailed information on the experimental setup can be found in Refs.^26,31^. The incident power on the sample was approximately 20 mW, and the irradiated area ranged from 5 to 15 μm, depending on the wavevector or angle of incidence. The frequency versus wavevector dispersion curves for phonons in the samples under study were determined by measuring the projection of incident light wavevector *q* (ranging from 4 to 23 μm^−1^) and the frequency shift *Δf* of the backscattered light^32^. Momentum conservation in the scattering process dictates that the wavevector of acoustic waves equals the projection of the incident light wavevector in the sample plane. Therefore, the wavevector *q* of the acoustic waves can be expressed as:

1 $$q=\frac{4\pi sin\theta }{{\lambda }_{0}}$$

Furthermore, the phase velocity (${v}_{SAW}$) of SAWs can be calculated using the following relation:

2 $${v}_{SAW}=\frac{\Delta {f}_{SAW}{\lambda }_{0}}{2\text{sin}\theta } =\frac{2\pi \Delta {f}_{SAW}}{q}$$

Here, $\Delta {f}_{SAW}$ represents the Brillouin frequency shift due to SAW, and $\theta$ is the incident angle of the laser beam (Fig. 1a).

## Results

The scattering of incident light in materials varies based on their properties. In transparent materials, light is inelastically scattered from bulk acoustic modes through an elasto-optic coupling mechanism. Conversely, in opaque materials, light is scattered from the surface of the material via a surface ripple mechanism known as surface Brillouin scattering. The periodic displacements caused by the propagation of SAWs create ripple-like structures on the surface, leading to information about the SAWs being contained in the scattering light^33^. Semi-opaque materials often exhibit both bulk and surface scattering, with the propagation of scattered light from each mechanism dependent on the material's opacity. Among various types of SAWs, Rayleigh waves typically exhibit linear dispersion^26,31,34–36^ in homogeneous materials, with their phase velocity always lower than the slowest transverse bulk wave velocity. However, this linear dispersion is not common in multilayer thin films. These films can be classified into *slow-on-fast* (when the velocity of the transverse bulk wave in the multilayer is smaller than that of the substrate) and *fast-on-slow* (vice versa) systems^37^.

The typical BLS spectra for the samples with different underlayer materials are illustrated in Fig. 1b–e, showing high-intensity peaks corresponding to Rayleigh waves (marked as R) and low-intensity peaks corresponding to Sezawa waves (identified from the FEM-based simulations and marked S in Fig. 1b–e). These Sezawa waves exist when the transverse bulk-wave velocity in the layer (${v}_{T}^{layer}$) is smaller than that in the substrate (${v}_{T}^{substrate}$) and only for a restricted range of $qh$, as discussed in the next section, where *h* represents the total thickness of the multilayer system.

**Fig. 2:**
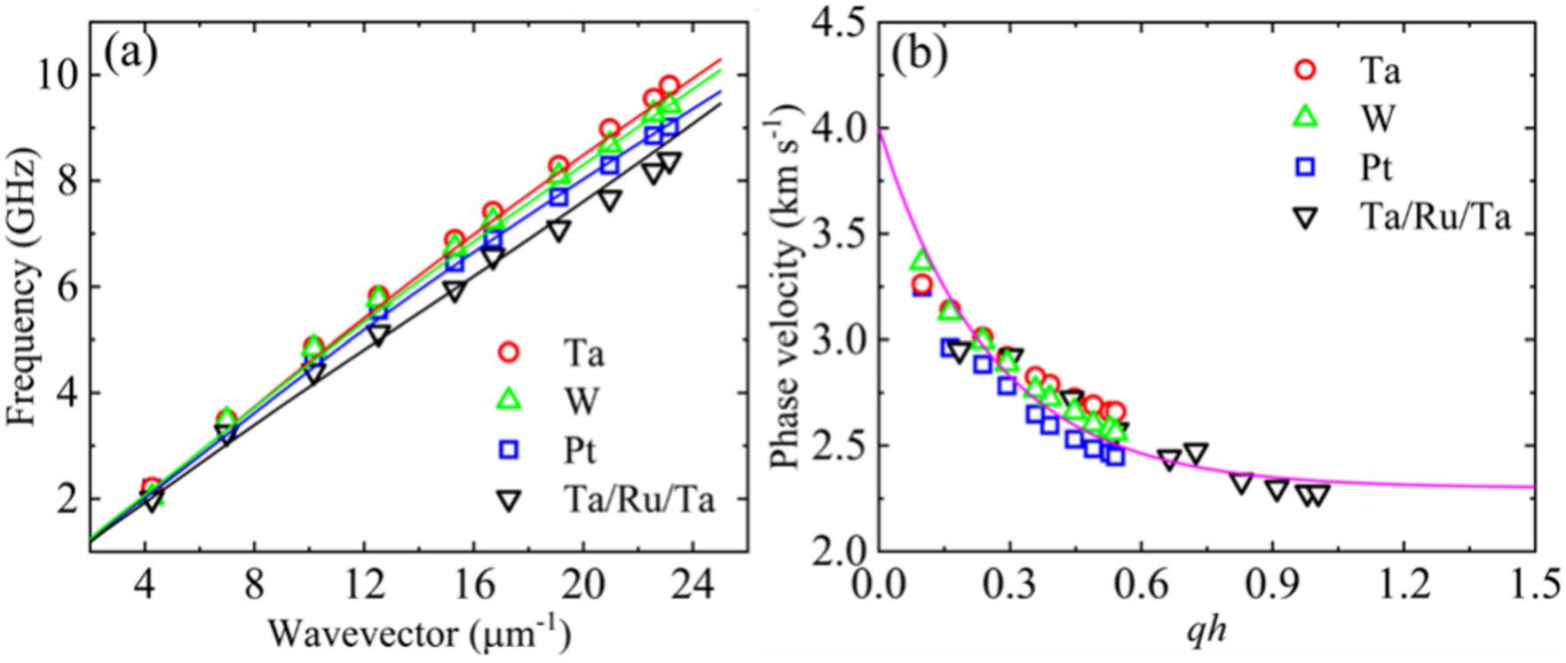
(**a**) Rayleigh wave frequency obtained from samples with different underlayer materials with varying wavevectors. The points represent the experimental data, and the lines represent the dispersions obtained from the finite element method-based simulations. (**b**) Phase velocity of Rayleigh SAWs with an uncertainty of 2% obtained from samples for different wavenumbers. The solid curve represents the fit to an exponential decay function.

Figure 2a shows the frequency versus wavevector dispersion of Rayleigh waves measured for different samples. Different underlayer materials have different densities (*ρ*), which can be compared as *ρ*_Ru_ < *ρ*_Ta_ < *ρ*_W_ < *ρ*_Pt_ (see Supplementary Information S3). The frequency versus wavevector dispersion of Rayleigh waves decreases with increasing underlayer density. Figure 2b shows the phase velocities, calculated using Eq. (2), as a function of *qh* (i.e., wavevector, *q* × thickness, *h*). Here, *h* is the total thickness of the multilayer system on top of the substrate, i.e., *h* = *t*_X_ + *t*_CoFeB_ + *t*_MgO_ + $t_{{{\text{Al}}_{{2}} {\text{O}}_{{3}} }}$, where *t*_X_, *t*_CoFeB_, *t*_MgO_, and $t_{{{\text{Al}}_{{2}} {\text{O}}_{{3}} }}$ are the thicknesses of the underlayer and the CoFeB, MgO and Al_2_O_3_ layers, respectively.

The phase velocity decreases exponentially with *qh,* indicating that the studied films can be classified as slow-on-fast systems. In these systems, the highest phase velocity (obtained as $qh\to 0$) equals the velocity of the transverse wave $\left( {v_{T} } \right)$ in the substrate (see Supplementary Table S1 for the transverse wave velocity in each layer). The phase velocity decreases asymptotically with *qh* and equals the Rayleigh wave velocity (${v}_{R}$) in the layer deposited on the substrate^33^. We extract the values of ${v}_{T}$ and ${v}_{R}$ in our studied samples as 4 km s^−1^ and 2.3 km s^−1^, respectively, by fitting the experimental data points with an exponential decay function. The extracted values of transverse wave velocity (${v}_{T}$) and Rayleigh wave velocity (${v}_{R}$) in the studied samples suggest that the stacked layers (i.e., underlayer/CoFeB/MgO/Al_2_O_3_) can be classified as effective layers, with the Rayleigh wave velocity calculated accordingly^32^. As the problem is not trivial, we perform FEM-based simulations to determine the velocities of SAWs for large *qh*, providing further insights into the behavior of the SAWs in the multilayer system, as described in “FEM simulation” section.

## FEM simulation

The FEM-based simulations were performed in COMSOL Multiphysics software within a 3D domain^38^. The unit cell chosen for the simulations comprised a long cuboid with dimensions 100 (x) nm × 100 (y) nm × 3723.4 (z) nm, where the thickness (z-direction) consisted of 10 nm of Al_2_O_3_, 2 nm of MgO, 1.4 nm of CoFeB, 10 nm of underlayer, 700 nm of SiO_2_ and 3000 nm of Si. The unit cell is constructed either as a multilayer (each individual layer) or an effective layer with uniform elastic properties. The substrate is a uniform elastic half-space with layer(s) of determined thickness on it. The simulation operates under the assumption that the layers are ideally flat and parallel and are perfectly bonded with zero interfacial thickness. Additionally, the layer possesses uniform thickness and uniform elastic properties throughout, with no interfacial roughness or defects^32^.

Further, the boundary conditions were applied using Bloch–Floquet periodic boundary conditions for each component of displacement on walls perpendicular to the free surface, with fixed boundary conditions for the wall opposite to the free surface to account for the exponential decay of the SAW amplitude:

3 $$ \begin{aligned} & a\;exp\left[ {i\left( {q_{x} x + q_{y} y} \right)} \right] \\ & b\;exp\left[ {i\left( {q_{x} x + q_{y} y} \right)} \right] \\ \end{aligned} $$

where $a$ and $b$ represent the components of the displacement in the x- and y-direction in the cartesian co-ordinate system and $q_{x}$ and $q_{y}$ are the wavevector components given as:

4 $$ \begin{aligned} q_{x} & = \left( {2\pi cos \alpha } \right)/\lambda_{SAW} \\ q_{y} & = \left( {2\pi cos \beta } \right)/\lambda_{SAW} \\ \end{aligned} $$

Here, $\alpha$ and $\beta$ are the angles between wavevector and x- and y-axes, respectively. ${\lambda }_{SAW}$ is the wavelength of SAW.

**Fig. 3:**
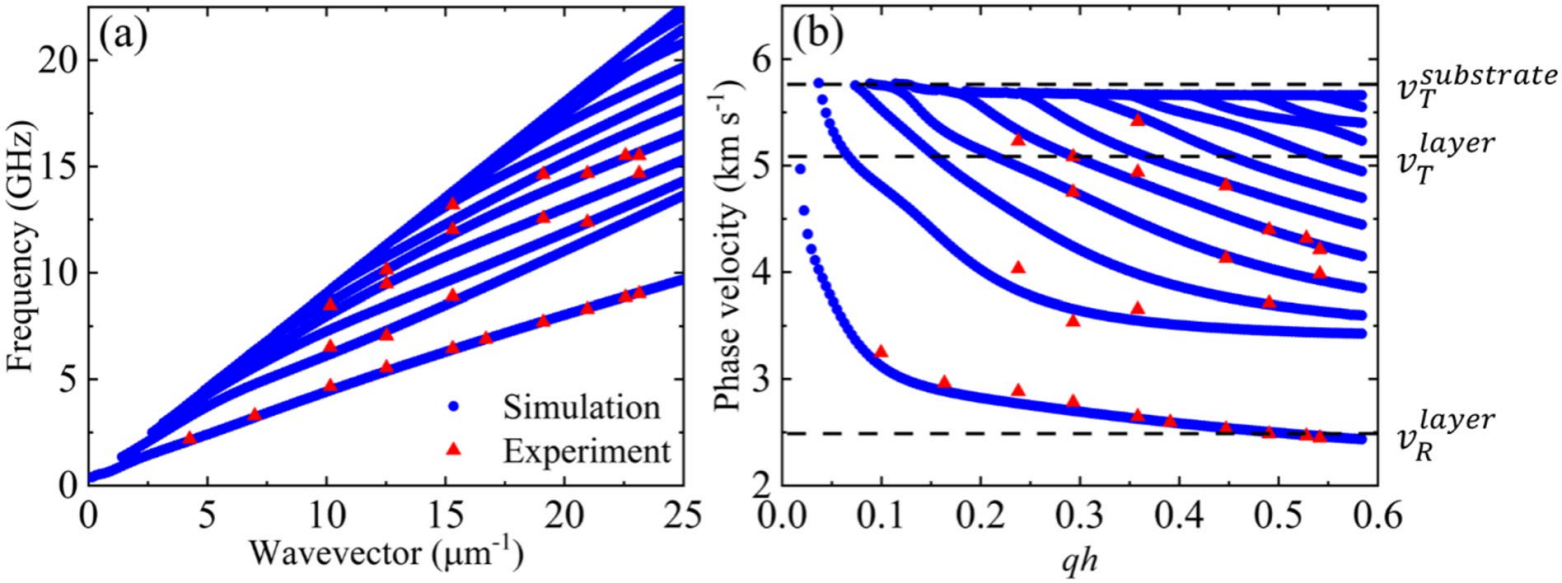
(**a**) Frequency vs. wavevector dispersion relation and (**b**) phase velocity dispersion of SAWs obtained from the Si/SiO_2_/Pt/CoFeB/MgO/Al_2_O_3_ sample. The red dots are the experimental results obtained from the BLS measurements, whereas the blue dots are the FEM simulation results.

Figure 3a shows the dispersion characteristics for Rayleigh and Sezawa waves (including higher-order Sezawa modes) obtained from the simulation (blue solid circles) and experiment (red solid triangles) for Si/SiO_2_/Pt/CoFeB/MgO/Al_2_O_3_. In the experiment, Rayleigh waves are visible, while low-intensity Sezawa waves are identified from the FEM simulations. In the simulations, we used the elastic constant and density of each layer from the literature (see Supplementary Table S3). The experimental results for Rayleigh waves and Sezawa waves from FEM simulations showed excellent agreement, especially for Rayleigh waves. It is important to note that the materials in the samples exhibit different crystallographic symmetries, impacting the parameters due to their specific characteristics and symmetry.

We also calculated the phase velocity of SAWs propagating in the Si/SiO_2_/Pt/CoFeB/MgO/Al_2_O_3_ sample and plotted it as a function of wavenumber, as shown in Fig. 3b. The error in the phase velocity for all the samples is within 2%. Here, the red points represent the experimental results, whereas the blue points represent the results obtained from FEM simulations. The phase velocity dispersion plots reconfirm that the studied multilayer on Si/SiO_2_ is a slow-on-fast system. For slow-on-fast systems, an elastically soft layer on an elastically hard substrate leads to a decrease in the Rayleigh SAW velocity and the formation of higher-order modes known as Sezawa waves. The simulated phase velocity dispersion graphs allow us to estimate the phase velocities of transverse bulk waves in the layer and in the substrate, which are approximately 5.06 km s^−1^ and 5.74 km s^−1^, respectively. The phase velocity of the Rayleigh SAW in the layer is estimated to be approximately 2.54 km s^−1^. Here, the substrate is Si/SiO_2,_ and the layer is Pt/CoFeB/MgO/Al_2_O_3_. These velocity estimates serve as the foundation for calculating the elastic parameters of the effective layers in all studied samples.

## Discussion

Further COMSOL simulations were performed to investigate the impact of the underlayer material on the velocity of the SAWs in the samples. The penetration depth of Rayleigh SAWs depends upon the wavevector and is greater than the thickness of the deposited multilayer (*h*). Thus, the deposited layers of X/CoFeB/MgO/Al_2_O_3_ can be treated as effective layers, and thus, the effective elastic parameters of the multilayers can be estimated. Various methods exist for this estimation^39,40^, with the proportion method being utilized in this case. The effective elastic parameter is calculated using the elastic parameter of individual layers. For example, consider the sample with Pt underlayer. This sample will consist of 10 nm of Pt, 1.4 nm of CoFeB, 2 nm of MgO and 10 nm of Al_2_O_3_ deposited on a Si/SiO_2_ substrate. Thus, the effective layer corresponding to this multilayer will have a total thickness of 23.4 nm with effective elastic parameter (EEP) calculated as:

5 $$EEP=\left({\sum }_{i=all\, layers}{t}_{i}*E{P}_{i}\right)/total\, thickness$$

**Fig. 4:**
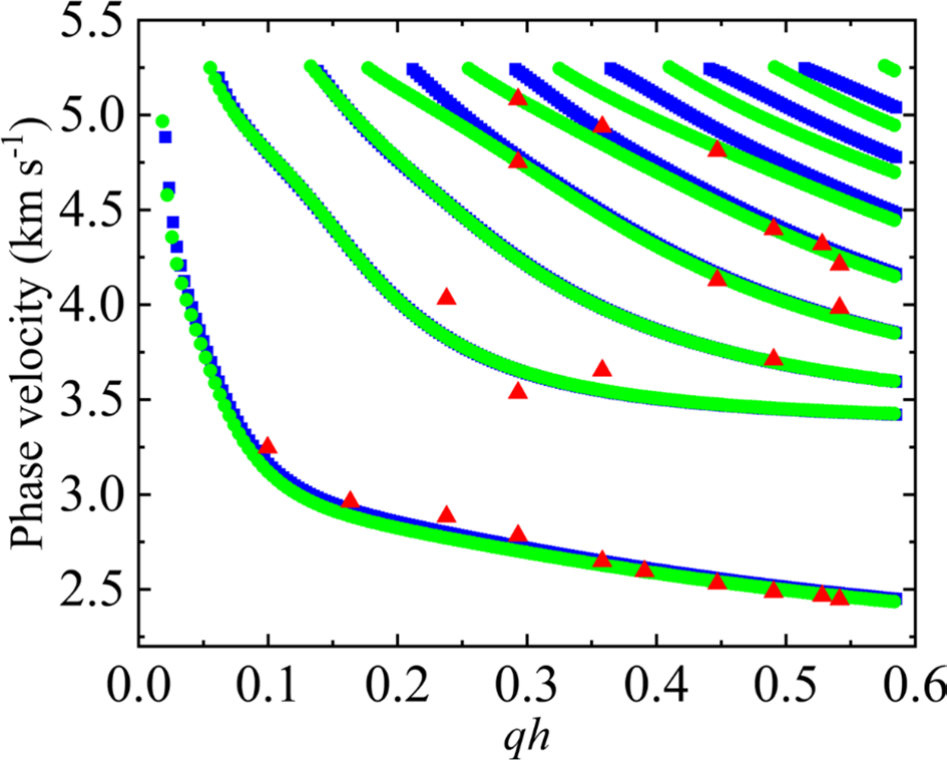
The phase velocity of SAWs obtained from the Si/SiO_2_/Pt/CoFeB/MgO/Al_2_O_3_ samples considering each layer as an individual layer (green, solid circles) and considering the whole layer as an effective layer (blue, solid square) as a function of *qh*. The red triangular points represent the phase velocity obtained from the experiment.

Here, ${t}_{i}$ is the thickness and $E{P}_{i}$ is the elastic parameter such as elastic constants and density of the ${i\text{th}}$ layer^41,42^. In addition to the simulation with the elastic parameters of individual layers, another simulation with these calculated effective elastic parameters is also performed and compared. Figure 4 shows the phase velocity dispersion of SAWs for both simulations. The simulation results comparing the elastic parameters of individual layers and effective layers show excellent agreement with the experimental data, validating this estimation approach.

**Fig. 5:**
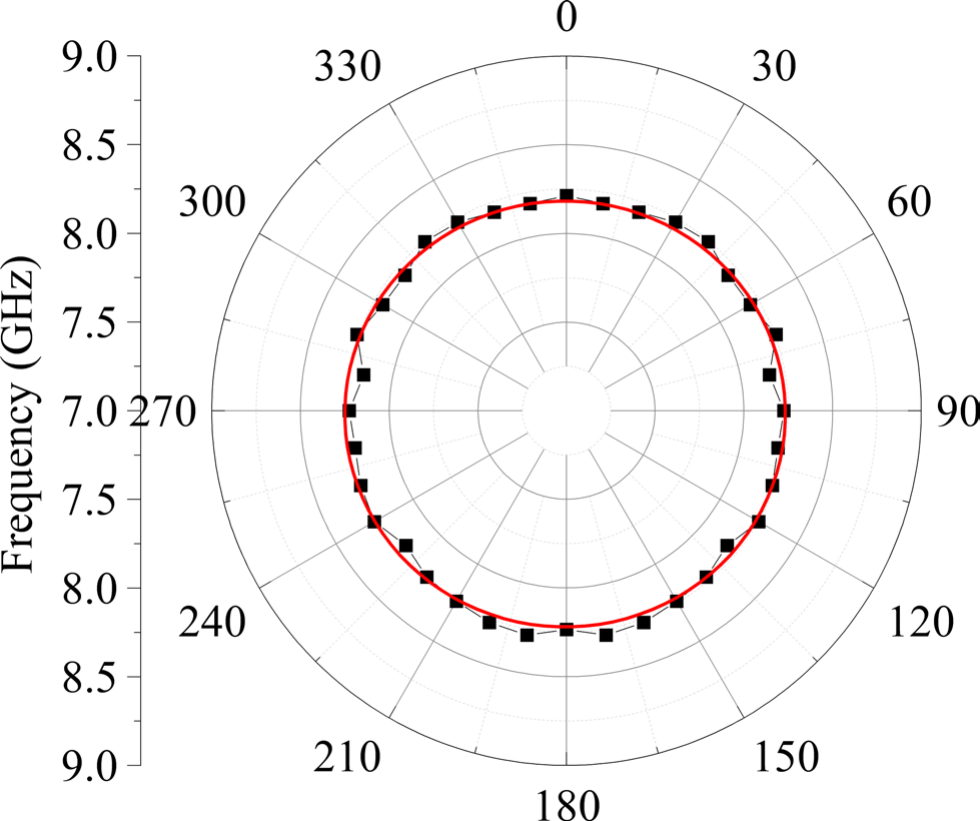
Angular dependent behavior of the Rayleigh wave frequency in the sample with a Pt underlayer measured for wavevector *q* = 20.76 μm^−1^.

Our multilayer films are composed of multiple layers exhibiting distinct crystallographic symmetries and unique elastic tensor components. These components can be effectively represented by elastic parameters such as Young’s modulus, Poisson’s ratio and density, especially when treating multilayer films as isotropic layers. The isotropic characteristics of the film surface, denoting its symmetry, can be elucidated by analyzing the angular dispersion of Rayleigh waves. Figure 5 shows the angular dispersion data obtained for the sample with a Pt underlayer and a laser incident angle $\theta = 61.5^\circ$ corresponding to the wavevector *q* = 20.76 μm^−1^. This observation confirms the isotropic nature of the surface. Even changing the underlayer material does not influence the isotropic nature of the surface in the samples. As the penetration depth of SAWs into the studied multilayers is greater than the thickness of the multilayers, SAWs exhibit isotropic behavior at the surface.

**Fig. 6:**
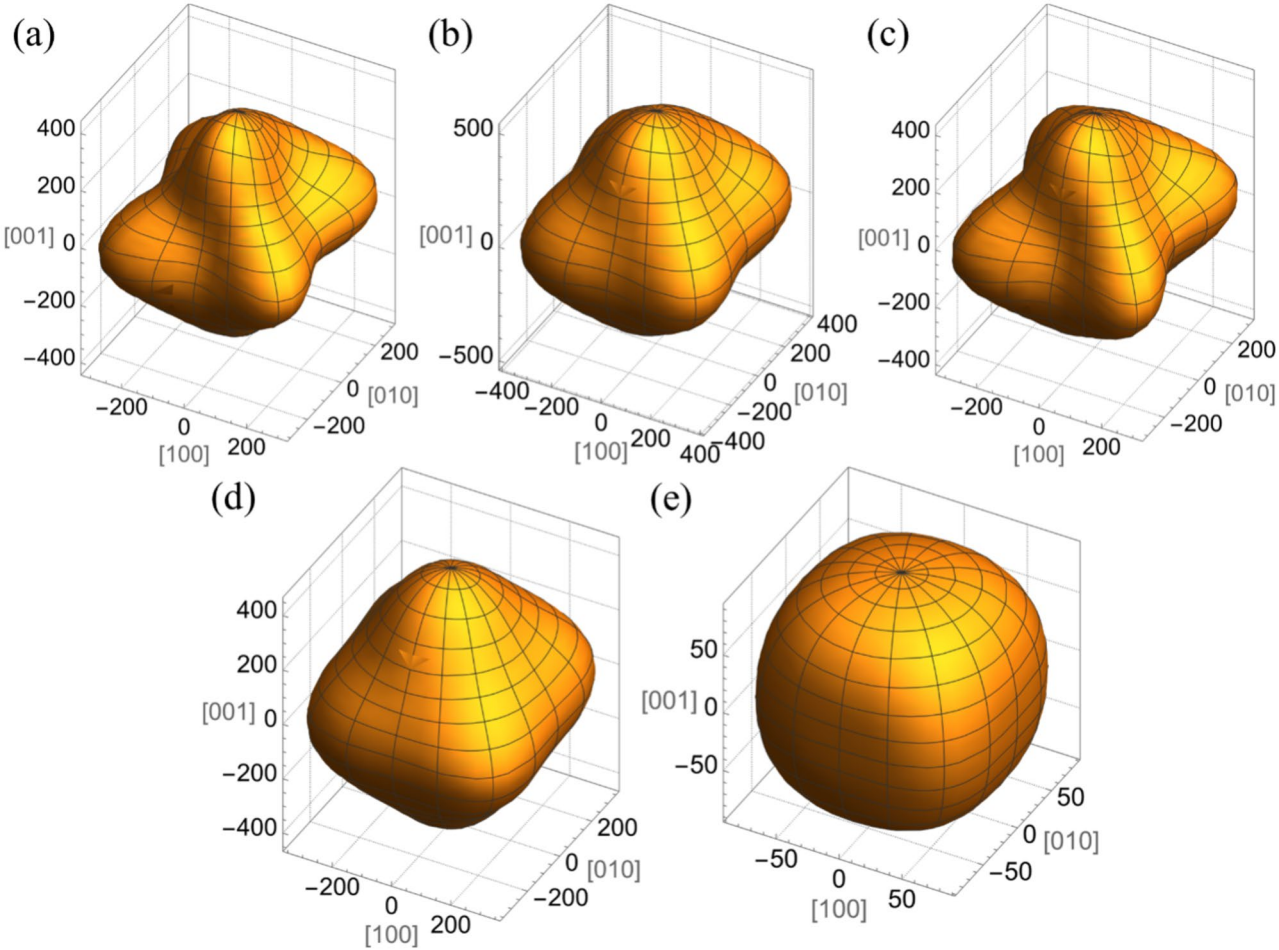
3D Young’s modulus (in GPa) of the different multilayers: (**a**) Ta/CoFeB/MgO/Al_2_O_3_, (**b**) W/CoFeB/MgO/Al_2_O_3_, (**c**) Pt/CoFeB/MgO/Al_2_O_3_, (**d**) Ta/Ru/Ta/CoFeB/MgO/Al_2_O_3_, and (**e**) Si/SiO_2_/Pt/CoFeB/MgO/Al_2_O_3_.

Using the proportion method, the effective elastic tensor was calculated to generate a 3D plot of Young’s modulus ($E$). This plot, depicted in Fig. 6, shows the spatial variation in Young’s modulus for the effective layers by treating the multilayers in the sample as a single effective layer. Figure 6a–d illustrates the spatial dependence of Young’s modulus for the effective layers with different underlying layers, while Fig. 6e focuses on Young’s modulus for a sample with a Pt underlayer on a Si/SiO_2_ substrate.

The Young’s modulus $E$ was determined using the generalized equation for anisotropic materials, derived from the elastic tensor $C$ calculated via the weighted average method. The compliance matrix $S$ was then obtained as the inverse of $C$. A unit vector (${\varvec{n}}$) representing the spatial direction is defined as:

6 $${\varvec{n}}=\text{cos}\left(\varphi \right)\text{sin}\left(\theta \right)\hat{i}+\text{sin}\left(\theta \right)*\text{sin}\left(\varphi \right)\hat{y}+\text{cos}\left(\theta \right)\hat{z}$$

Subsequently, the Young’s modulus $E$(${\varvec{n}}$, $S$) was calculated and plotted using the following formula^43^:

7 $$E\left({\varvec{n}},{\mathbb{S}}\right)={\left({\mathbb{S}}\cdot {{\varvec{n}}}^{\otimes 4}\right)}^{-1}={\left({S}_{ijkl}{n}_{i}{n}_{j}{n}_{k}{n}_{l}\right)}^{-1}={\left({S}_{ijkl}{N}_{ij}{N}_{kl}\right)}^{-1}={\left({\mathcal{N}}^{T}\mathcal{S}\mathcal{N}\right)}^{-1}$$

Here, ${N}_{ij}={n}_{i}{n}_{j}$, $\mathcal{N}$ and $\mathcal{S}$ denote the vector and matrix representation of the tensors in normalized Voigt notation.

The 3D Young’s modulus of the effective layers with different underlying layers (Fig. 6a–d) displays anisotropy due to the presence of the anisotropic Al_2_O_3_ layer. In contrast, the overall sample exhibits isotropic behavior, as evidenced by Figs. 5 and 6e, attributed to the high thickness of the Si/SiO_2_ layer and the isotropic nature of each material, as confirmed by the proportion technique.

Despite the thinness of our multilayers in comparison to the typical penetration depth of surface acoustic waves (SAWs) in opaque materials (defined as two wavelengths), the transparency of our materials implies a potentially greater SAW penetration depth. This likely accounts for the observed isotropic behavior in SAW propagation, where the substrate's influence predominates. These findings are particularly relevant to phonon‒magnon interactions, as the presence of a magnetic layer within the multilayer can impact the sensitivity of the interaction to anisotropy. However, further investigation is necessary to fully explore this effect.

## Conclusion

Using Brillouin light scattering, we investigated thermally excited surface acoustic waves (SAWs) in Si/SiO_2_/X/CoFeB/MgO/Al_2_O_3_ heterostructures with different underlayer materials (Pt, W, Ta, and Ta/Ru/Ta). Thermal phonons were detected in the backscattering geometry to analyze the dispersion of SAWs, including Rayleigh and Sezawa waves, with frequencies reaching up to a few GHz and varying wavevectors up to 23 µm^−1^. The experimental findings were validated through finite element method simulations. Additionally, we determined the effective elastic parameters, such as the elastic tensor and density. The Young’s modulus and Poisson’s ratio of the multilayer were calculated for different underlayer materials by treating the deposited multilayer on Si/SiO_2_ as a unified layer. These extracted elastic parameters are expected to be valuable for comprehending and controlling the interaction of phonons with other quasiparticles or spin textures in similar heterostructures.

### Supplementary Information

Supplementary Information.

## Data Availability

The datasets generated during and/or analyzed during the current study are available in the Zenodo at 10.5281/zenodo.10909449.
