# Supplementary material for: Effect of the underlayer on the elastic parameters of the CoFeB/MgO heterostructures
Source: Sci Rep. 2024 Aug 31;14:20259. doi: 10.1038/s41598-024-71110-1 (PMC11365987; doi:10.1038/s41598-024-71110-1)
Supplement: Supplementary file 1 — Supplementary Information. [file 41598_2024_71110_MOESM1_ESM.docx]

**Supplementary for Effect of the underlayer on the elastic parameters of the CoFeB/MgO heterostructures**

S. Shekhar^1*^, S. Mielcarek^1^, Y. Otani^2,3^, B. Rana^1^, A. Trzaskowska^1^

^1^*Institute of Spintronics and Quantum Information, Faculty of Physics, Adam Mickiewicz University, Uniwersytetu Poznańskiego 2, 61-614 Poznan, Poland*

^2^*Center for Emergent Matter Science, RIKEN, 2-1 Hirosawa, Wako 351-0198, Japan*

^3^*Institute for Solid State Physics, University of Tokyo, Kashiwa, Chiba 277-8581, Japan*

Email: [shashank.shekhar@amu.edu.pl](mailto:Shashank.shekhar@amu.edu.pl)

**S1. Transverse wave velocity**

The transverse wave velocity for each layer can be calculated from the known $C_{44}$ component of the elastic tensor using the formula $v_{T}=\sqrt{C_{44}/\rho}$. The calculated values of $v_{T}$ are tabulated below:

**Table S1.** Transverse wave velocity in different layers in [001] direction

| Different layers | Transverse wave velocity (m s^-1^) in [001] direction |
| --- | --- |
| Si | 5857 |
| SiO_2_ | 3365 |
| Ta | 2009 |
| Pt | 1882 |
| W | 2888 |
| Ru | 3852 |
| CoFeB | 2541 |
| MgO | 6559 |
| Al_2_O_3_ | 6825 |

**S2. Lattice parameter**

The lattice parameter for all the layers is given below.

**Table S2.** Lattice parameter of individual layers

|  | Lattice parameter(s) (in $Å$) [1] |
| --- | --- |
| Si | 5.43 |
| SiO_2_ | a = 4.91, c = 5.40 |
| Ta | 3.3 |
| Pt | 3.92 |
| W | 3.17 |
| Ru | a = 2.71, c = 4.28 |
| CoFeB | 2.8 |
| MgO | 4.21 |
| Al_2_O_3_ | a = 4.81, c = 13.12 |

**S3. FEM simulation**

We used FEM based simulation to study thermally generated SAWs in COMSOL Multiphysics software. The elastic parameters such as elastic tensor and density for each material is taken from the references indicated below in the Table S3.

**Table S3.** Elastic tensor components (*c_ij_*) in GPa and density ($\rho$) in kg m^-3^ of materials are used for the simulation.

|  | Si[2] | SiO_2_[3] | Ta[4] | W[4] | Pt[5] | Ru[6] | CoFeB[7] | MgO[8] | Al_2_O_3_[9] |
| --- | --- | --- | --- | --- | --- | --- | --- | --- | --- |
| $\rho$ | 2332 | 2650 | 16600 | 19300 | 21450 | 12200 | 6970 | 3580 | 3950 |
| c_11_ | 166 | 75 | 261 | 523 | 347 | 563 | 210 | 296 | 466 |
| c_12_ | 64 | 15 | 157 | 204 | 251 | 188 | 130 | 95 | 127 |
| c_13_ | 64 | 15 | 157 | 204 | 251 | 168 | 130 | 95 | 117 |
| c_14_ | 0 | 0 | 0 | 0 | 0 | 0 | 0 | 0 | 94 |
| c_22_ | 166 | 75 | 261 | 523 | 347 | 563 | 210 | 296 | 466 |
| c_23_ | 64 | 15 | 157 | 204 | 251 | 168 | 130 | 95 | 117 |
| c_24_ | 0 | 0 | 0 | 0 | 0 | 0 | 0 | 0 | -94 |
| c_33_ | 166 | 75 | 261 | 523 | 347 | 624 | 210 | 296 | 506 |
| c_44_ | 80 | 30 | 82 | 161 | 76 | 181 | 45 | 154 | 235 |
| c_55_ | 80 | 30 | 82 | 161 | 76 | 181 | 45 | 154 | 235 |
| c_56_ | 0 | 0 | 0 | 0 | 0 | 0 | 0 | 0 | 94 |
| c_66_ | 80 | 30 | 82 | 161 | 76 | 191 | 45 | 154 | 170 |

**S4. Elastic tensor of the effective layer**

The elastic tensor for the effective layer is calculated using weighted average method and is given in table S4. The calculated elastic tensor suggest that the examined samples have (or close to) trigonal symmetry.

**Table S4.** Calculated Elastic tensor components (*c_ij_*) in GPa and density ($\rho$) in kg m^-3^ of effective layer.

|  | Ta underlayer | W underlayer | Pt underlayer | Ta/Ru/Ta underlayer |
| --- | --- | --- | --- | --- |
| $\rho$ | 9505 | 10659 | 11578 | 10747 |
| c_11_ | 361 | 461 | 385 | 447 |
| c_12_ | 175 | 195 | 215 | 181 |
| c_13_ | 82 | 115 | 135 | 129 |
| c_14_ | -2 | -2 | -2 | -1 |
| c_22_ | 338 | 450 | 375 | 449 |
| c_23_ | 112 | 131 | 151 | 131 |
| c_24_ | 24 | 24 | 24 | 13 |
| c_33_ | 432 | 531 | 455 | 520 |
| c_34_ | -16 | -16 | -16 | -9 |
| c_44_ | 123 | 164 | 127 | 146 |
| c_46_ | 6 | 0 | 0 | 0 |
| c_55_ | 84 | 131 | 95 | 132 |
| c_56_ | 14 | 14 | 14 | 11 |
| c_66_ | 171 | 211 | 175 | 179 |

**S6. Young’s modulus and Poisson’s ratio using ELATE software**

The Young’s modulus and Poisson's ratio for the effective layers were extracted and plotted using ELATE [10], an open-source online tool tailored for analysing elastic tensors. The estimated values are summarized in Table 1 and the plot of Poisson’s ratio is shown in Fig. S1.

**Table S5.** Young’s modulus, Poisson’s ratio, and density of uniform effective layers along [001] (isotropic)

| Underlayer material | Density (kg m^-3^) | Young’s modulus (GPa) | Poisson’s ratio |
| --- | --- | --- | --- |
| Ta | 9505 | 249 | 0.31 |
| W | 10659 | 393 | 0.24 |
| Pt | 11578 | 293 | 0.29 |
| Ta/Ru/Ta | 10747 | 375 | 0.26 |


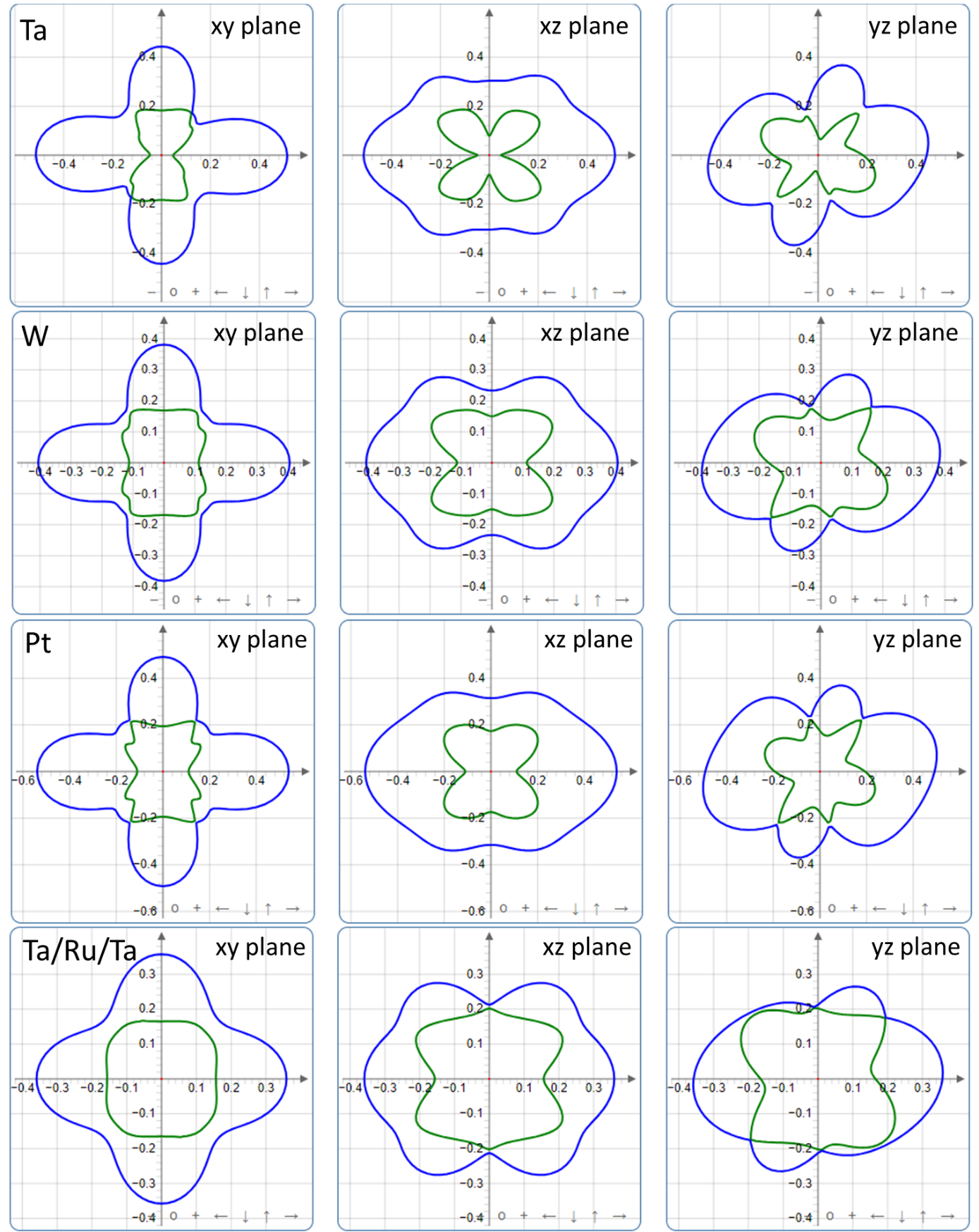


***Fig. S1.*** *Spatial plot of Poisson’s ratio for sample X/CoFeB/MgO/Al_2_O_3_ with X indicate different underlayer material.*

**References**

[1] MatWeb, (n.d.). https://www.matweb.com/ (accessed December 17, 2023).

[2] V. Kaajakari, Silicon as an anisotropic mechanical material - a tutorial, (2023). http://www.kaajakari.net/~ville/research/tutorials/elasticity_tutorial.pdf (accessed April 3, 2023).

[3] G. Carlotti, L. Doucet, M. Dupeux, Elastic properties of silicon dioxide films deposited by chemical vapour deposition from tetraethylorthosilicate, Thin Solid Films 296 (1997) 102–105. doi: 10.1016/S0040-6090(96)09346-7.

[4] F.H. Featherston, J.R. Neighbours, Elastic Constants of Tantalum, Tungsten, and Molybdenum, Physical Review 130 (1963) 1324. doi: 10.1103/PhysRev.130.1324.

[5] R.E. Macfarlane, J.A. Rayne, C.K. Jones, R.E. Macfarlane, J.A. Rayne, C.K. Jones, Anomalous temperature dependence of shear modulus c_44_ for platinum, PhL. 18 (1965) 91–92. doi: 10.1016/0031-9163(65)90659-1.

[6] R. Ramji Rao, J.V.S.S. Narayana Murthy, Lattice Dynamics and Thermal Expansion of Ruthenium, Zeitschrift Fur Naturforschung - Section A Journal of Physical Sciences 34 (1979) 724–730. doi: 10.1515/ZNA-1979-0609/MACHINEREADABLECITATION/RIS.

[7] S. Shekhar, S. Mielcarek, Y. Otani, B. Rana, A. Trzaskowska, Influence of CoFeB layer thickness on elastic parameters in CoFeB/MgO heterostructures, Sci. Rep. 13 (2023) 1–11. doi: 10.1038/s41598-023-37808-4.

[8] S. Tsuboi, K. Adachi, A. Nagakubo, al -, G.-S. Wang, E. Krisztina Delczeg-Czirjak, Q.-M. Hu, K. Marklund, S.A. Mahmoudl, S.A. Mahmoud, Elastic Constants of Magnesium Oxide, Phys. Scr. 3 (1971) 75. doi: 10.1088/0031-8949/3/2/005.

[9] R.V.G.S. Rao, Elastic constants of alumina, Proceedings of the Indian Academy of Sciences - Section A 29 (1949) 352–360. doi: 10.1007/BF03172465/METRICS.

[10] R. Gaillac, P. Pullumbi, F.X. Coudert, ELATE: an open-source online application for analysis and visualization of elastic tensors, Journal of Physics: Condensed Matter 28 (2016) 275201. doi: 10.1088/0953-8984/28/27/275201.
